# Study of the Phytochemical Composition, the Antioxidant and the Anti-Inflammatory Effects of Two Sub-Saharan Plants: *Piliostigma reticulatum* and *Piliostigma thonningii*

**DOI:** 10.1155/2021/5549478

**Published:** 2021-05-04

**Authors:** K. Boualam, B. Ndiaye, H. Harhar, M. Tabyaoui, N. Ayessou, K. Taghzouti

**Affiliations:** ^1^Laboratory of Materials, Nanotechnology and Environment, Faculty of Sciences-Rabat, Mohammed V University of Rabat, BP 1014-Rabat, Morocco; ^2^Genomics of Human Pathologies Research Center, Faculty of Sciences, Mohammed V University in Rabat, BP1014 Rabat, Morocco; ^3^Electrochemistry and Membrane Processes Laboratory LEPM, Polytechnics School, Cheikh Anta Diop University, PB 5085 Dakar, Senegal

## Abstract

The aim of this study is to perform phytochemical screening of the leaves of *Piliostigma reticulatum* and *Piliostigma thonningii*, to determine the phenolic, flavonoids, tannins, and sugars content in their methanolic extracts, evaluate their antioxidant activity using the DPPH and the ABTS tests, and test their anti-inflammatory effect in vitro using the heat-induced albumin denaturation inhibition method. Phytochemical screening revealed the presence of polyphenols and alkaloids in the leaves of both plants. Yields of the extracts in this study ranged from 7% to 18% for *P. reticulatum* and 4% to 16% for *P. thonningii*. The phenolic content in the methanolic extract of *P. reticulatum* is 74.66 ± 1.76 *μ*g GAE/mL, which is significantly higher than that of *P. thonningii* (56.54 ± 1.24 *μ*g GAE/mL). Both plants showed good antioxidant activity. In fact, for the DPPH test, the IC50 value is 8.88 ± 0.11 *μ*g/mL for *P. reticulatum* and 17.64 ± 0.68 *μ*g/mL for *P. thonningii*. For the ABTS assay, the IC_50_ values of the two plants are, respectively, 9.78 ± 1.83 *μ*g/mL and 13.47 ± 2.62 *μ*g/ml, statistically comparable and significantly higher than the IC_50_ of the standard 30.76 ± 0.18 *μ*g/ml. Leaf extracts from both plants were effective against heat-induced denaturation of albumin. The activity of *P. reticulatum* is indeed comparable to that of the standard with an IC_50_ value of 121.43 ± 1.55 *μ*g/mL and higher than that of *P. thonningii* with an IC_50_ value of 170.15 ± 1.09 *μ*g/mL. These results show that both plants exhibit significant antioxidant and anti-inflammatory activities. Therefore, their chemical compounds could have potential applications as antioxidant and anti-inflammatory drugs.

## 1. Introduction

Oxidative stress occurs when cells undergo an excessive production of oxygenated free radicals that exceeds their antioxidant power [1]. Oxidative stress can lead to inflammation in many situations such as excess temperature and radiation but it is often its consequence [2]. Oxygenated free radicals are mediators of inflammation that intervene during phagocytosis or to maintain the inflammatory state [3–5]. Indeed, the superoxide ion (O_2_^−^) is implicated in the proliferation of fibroblasts, oxygen peroxide (H_2_O_2_) acts in the activation of transcription factors such as NFkB, and nitric oxide (NO) intervenes in the regulation of vascular tone [6]. The close relationship between oxidative stress and inflammation may explain why molecules with an antioxidant effect make good candidates for anti-inflammatory treatments [7]. The interest of this study in anti-inflammatory activity is due to the fact that inflammation is an essential component of all chronic diseases as there is increasing evidence suggesting that excessive inflammation has a great impact on the physiopathology of stress-related diseases, which today constitutes 75% to 90% of human pathologies [8]. Since ancient times, plants have played a significant role in the treatment of many diseases. In this study, we aimed to compare the antioxidant and anti-inflammatory activities of two very similar and widely used Sub-Saharan tries: *Piliostigma reticulatum* and *Piliostigma thonningii*. These plants belong to the Caesalpinia family and are present throughout the area between Senegal and Sudan [9]. Morphologically, these two shrubs attain 5–10 m height [10], look alike, and can easily be confused especially in Senegal; they have the same vernacular name “Nguiguis” [11]. However, *P. thonningii* can be distinguished by a ferruginous pubescence below the pods and leaves, which are less split and larger than those of *P. reticulatum* [8–12]. In traditional medicine, all parts of these shrubs are used. The leaves contain very similar essential oils with an antibacterial effect [8], and their use in infusion or decoction is very effective against several diseases with an inflammatory background such as abdominal pain, rheumatism, headaches, and injuries [10]. In the present study, for the phenols and sugars content of the leaves of these two plants, the antioxidant and anti-inflammatory effects of their methanol extracts were assessed in order to elucidate their therapeutic properties.

## 2. Materials and Methods

### 2.1. Preparation of Extracts

The leaves of *Piliostigma reticulatum* and *Piliostigma thonningii* were collected at the Botanical garden of Cheikh Anta Diop University in Dakar. They were dried under shade for two weeks, powdered, then weighed for extraction. We used different solvents with increasing polarity: petroleum ether (PE), dichloromethane (DCM), dichloromethane/methanol (80/20), and methanol. 20 g of each plant was macerated with 200 mL of each solvent for four days at room temperature with continuous agitation. The obtained extracts were filtered then concentrated to dryness under pressure in a rotary evaporator. The crude extracts were stored at 4°C for further use.

### 2.2. Preliminary Phytochemical Screening

Qualitative tests protocols previously described by Kwaji et al. [13, 14] were used to detect the presence or the absence of alkaloids (Dragendorff, Mayer, and Wagner reagent), flavonoids (Mg-HCL test), tannins (ferric chloride reagent), terpenoides (chloroform and concentrated HSO_4_), sterols (reaction of Liebermann), and saponosides.

### 2.3. Determination of Total Phenolic Content (TPC)

The methanol leaf extracts of the two plants were used to determine the concentration of the phenolic content following the protocol of Folin Ciocalteu assay [15] with slight modifications. A solution of the crude extract diluted in methanol was prepared at a concentration of 1000 *μ*g/mL, from which two other dilutions were prepared: 500 *μ*g/mL and 250 *μ*g/mL. 0. 5 mL of each concentration was mixed with 2.5 mL of Folin–Ciocalteu reagent (10%) and 4 mL of Na_2_CO_3_ (7.5%). After 30 min of incubation at 45°C, the absorbance was measured at 765 nm. Values were compared to a blank sample. Results were expressed as gallic acid equivalent (GAE) in *μ*g/mL. The standard curve was drawn by plotting the absorbance against the concentration of gallic acid. The samples were analyzed in triplicate.

### 2.4. Determination of Total Flavonoid Content (TFC)

The content of flavonoids was determined by a colorimetric method using aluminum chloride [15]. 0.5 mL of diluted methanolic extract of each plant (1 mg/mL) was mixed with 3.2 mL of distilled water and 150 *μ*L of NaNO_2_ (5%). After 5 min, 150 *μ*L of AlCl_3_ (10%) was added, and then after 6 min, 1 mL of NaOH (1 M) was added. After 30 min of incubation at room temperature, the absorbance of the solution was read at 510 nm. Quantification of TFC was based on the standard curve of Quercetin solution. Flavonoids content was expressed as the Quercetin equivalent in *μ*g per 1 mL of extract. The samples were analyzed in triplicate.

### 2.5. Determination of Total Tannins Content (TTC)

The modified Broadhurst and Jones method [15] was followed to determine the Tannins Content in both plants' methanol leaves extracts, using catechin as a reference compound. 100 *μ*L of extract (1 mg/mL) was added to 3 mL of a solution of vanillin (4% in methanol) and 1.5 mL of concentrated HCl. After 2 min of incubation in obscurity, the absorbance was read at 500 nm. The TTC was calculated by a calibration curve of catechin. Tannins content was expressed as the catechin equivalent in *μ*g per 1 mL of extract. The sample was analyzed in triplicate.

### 2.6. Determination of Total Sugars Content (TSC)

The sulfuric acid-phenol method [16] was used to determine the total sugar content in *P. reticulatum* and *P. thonningii* methanol leaves extracts. 1 mL of the extract (1 mL/mg) was added to 1 mL of phenol (5%), and then we added 5 mL of concentrated sulfuric acid (H_2_SO_4_). After 10 min of agitation and 30 min of incubation at 30°C, the absorbance was read at 488 nm. The TSC was determined using a calibration curve of glucose. The sugars content was expressed as the *G* equivalent in *μ*g per 1 mL of extract. The sample was analyzed in triplicate.

### 2.7. Antioxidant Activity

#### 2.7.1. DPPH Test

The antiradical activity of both extracts was tested using the 2.2-diphenyl-1-picryl-hydrazyl (DPPH) with ascorbic acid as a positive control [17]. 0.5 mL of DPPH (79 *μ*g/mL) was added to a concentration range of extract (5–100 *μ*g/mL). The reaction was then incubated for 30 min in obscurity at room temperature. When DPPH is reduced by an antioxidant molecule, its violet color turns yellow. The absorbance was read at 517 nm and the scavenging activity was estimated in percentage using the following equation:

(1) $$\begin{array}{c} \text{Free radical scavenging}\left(\%\right)=\frac{\left(O.D\text{ control}-O.D\text{ test}\right)\;}{\left.O.D\text{ control}\right)}*100. \end{array}$$

IC_50_ was estimated using the graph of free radical scavenging against extract and standard concentrations. The concentrations were tested three times and the average was calculated.

#### 2.7.2. ABTS Essay

The test is based on the ability of an antioxidant to stabilize the blue-green colored cationic radical ABTS^+^ (2,2- azinobis-3-ethylbenzothiazoline-6-sulfate) by trapping a proton and transforming it into colorless ABTS^+^ [18].

A solution of ABTS (7 Mm) was prepared by mixing 72 mg of ABTS with 13.24 mg of potassium persulfate (2.45 mM) in 20 mL of distilled water. The mixture was incubated for 16 hours in the dark. The ABTS-+ solution (7 mM) was diluted with distilled water to reach an absorbance of 0.7 ± 0.02 at 734 nm. 1.9 mL of this solution has been added to 600 *μ*L of extract solution at different concentrations (5–100 *μ*g/mL) [19]. After incubation for 7 min at room temperature, the absorbance was measured at 734 nm.

The blank was prepared by replacing the extract solution with methanol. The scavenging activity was estimated in percentage using the following equation:

(2) $$\begin{array}{c} \text{Free radical scavenging}\left(\%\right)=\frac{\left(O.D\text{ control}-O.D\text{ test}\right)\;}{\left.O.D\text{ control}\right)}*100. \end{array}$$

IC_50_ was estimated using the graph of free radical scavenging against extract and standard concentrations. The concentrations were tested three times and the average was calculated.

### 2.8. Anti-Inflammatory Activity

To assess the anti-inflammatory activity of *P. reticulatum* and *P. thonningii* leaves, the inhibition of heat-induced protein denaturation protocol [20, 21] was followed with minor modifications. Diclofenac sodium was used as the reference drug. 0.05 mL of various concentrations (40 *μ*g/mL to 1000 *μ*g/mL) of extracts and the reference was mixed with 0.45 mL of bovine albumin (BSA) (5%) and the pH was fixed at 6.3 with 1 N HCl.

The mixture tubes were incubated at 37°C for 20 min; then, the temperature was raised to 57°C for 20 min. 2.5 mL of phosphate buffer solution (PBS) (pH = 6.3) was added to each reaction tube and the absorbance was measured at 660 nm. The control test tube contained distilled water and BSA; the product control test tube contained distilled water and the extract. The percentage of inhibition was calculated using the following formula:

(3) $$\begin{array}{c} \text{The percentage of inhibition}\left(\%\right)=\frac{\left(O.D\text{ control}-O.D\text{ test}\right)\;}{\left.O.D\text{ control}\right)}*100. \end{array}$$

All the concentrations were tested three times, and the average was used.

### 2.9. Statistical Study

The total phenolic-sugar contents, antioxidant, and in vitro anti-inflammatory activities assay results were shown as means ± SD of triplicate readings (*n* = 3). Data were analyzed with GraphPad Prism 6 version 6.01 and evaluated using an unpaired *t*-test and one-way analysis of variance ANOVA followed by Tukey's multiple comparisons test. The differences were regarded as statistically significant at *p* < 0.05.

## 3. Results and Discussion

### 3.1. Yields and Phytochemical Screening

**Figure 1:**
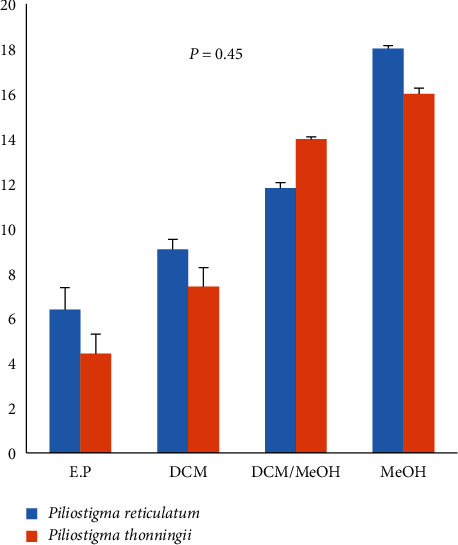
Extraction yields of both plants by different solvents. Statistically, the difference in yields between the two plants for each solvent is not significant (*p*=0.45 > 0.05).

Four extracts with different colors and aspects were obtained for each plant. Extraction yields are reported in Figure 1. The yields ranges are from 7% to 18% for *P. reticulatum* and 4% to 16% for *P. thonningii*. For each solvent, there is no significant difference between the yields of the two plants extracts. The higher the polarity of the solvents is, the higher the yields of the extracts are. Therefore, the yield of the methanolic extract is more important for both plants.

Preliminary phytochemical screening results are grouped in Table 1. Both *P. reticulatum* and *P. thonningii* leaves contain polyphenols, tannins, flavonoids, and alkaloids. Steroids were only found in Petroleum Ether and dichloromethane *P. thonningii* leaves extract, while DCM/methanol and DCM extracts showed the presence of terpenoids in *P. reticulatum*. Only *P. thonningii* leaves contain saponins. Extracts with the solvents DCM/MeOH and MeOH have a higher concentration of phytochemicals compared to other solvents.

### 3.2. Total Phenol, Flavonoids, Tannins, and Sugars Content

Given the fact that phytochemical compounds are more concentrated in methanol extracts, we chose to use them to continue our study. Total phenol content (TPC) estimated from the calibration curve of gallic acid showed that the methanol extracts of the two plants *P. reticulatum* and *P. thonningii* contain 74.66 ± 1.76 GAE *μ*g/mL and 56.54 ± 1.24 GAE *μ*g/mL, respectively. Total flavonoids (TFC), calculated using quercetin as a standard, showed that the concentration of flavonoids in *P. thonningii* methanol leaf extract is high (21. 59 ± 0. 75 QE *μ*g/mL) comparing to *P. reticulatum* methanol leaf extract (13.57 ± 0. 92 QE *μ*g/mL). According to the calibration curve of catechin, *P. reticulatum* methanol leaf extract contains more tannin (58.29 ± 0.94 CE *μ*g/mL) than the *P. thonningii* extract (33.87 ± 1.09 CE *μ*g/mL). The concentration of total sugars in *P. reticulatum* methanol leaf extract is high (71.79 ± 2.57 GE*μ*g/mL) comparing to *P. thonningii* methanol leaf (13.34 ± 1.82 GE*μ*g/mL) (Table 2).

### 3.3. Antioxidant Activity

#### 3.3.1. DPPH Test

**Figure 2:**
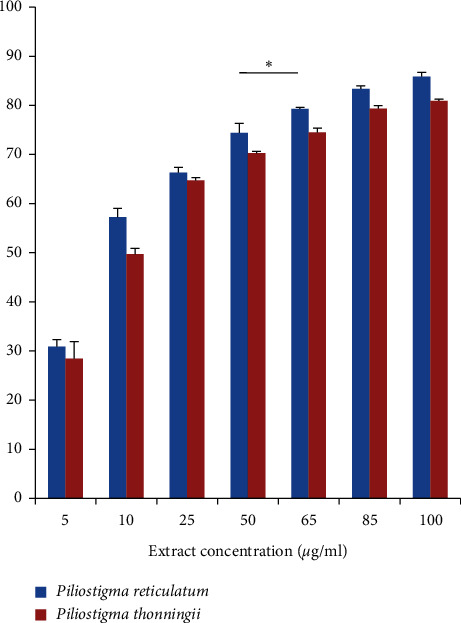
DPPH scavenging ability of *P. reticulatum* and *P. thonningii* methanolic leaves extracts. The kinetics of the two extracts is significantly different (*p* < 0.05).

The DPPH scavenging ability results of *P. reticulatum* and *P. thonningii* leaves are presented in Figure 2. For both plants, a DPPH reduction was observed starting from the smallest concentrations. At 10 *μ*g/mL, the methanol leaves' extracts of *P. reticulatum* and *P. thonningii* start showing a significant scavenging ability, which reached 57.25% and 49.75%, respectively. The inhibition kept rising up to the concentration of 100 *μ*g/mL reaching 85.89% for *P. reticulatum* and 80.98% for *P. thonningii*. The IC_50_ values for *P. reticulatum* and *P. thonningii* methanol leaves extracts were 8.88 ± 0.11 *μ*g/mL and 17.64 ± 0.68 *μ*g/mL, respectively. These values are statistically low, compared to the ascorbic acid IC_50_ (1.74 ± 0.28 *μ*g/mL) (Table 3). These results do not contradict however with the fact that both plants have a very high antioxidant effect.

#### 3.3.2. ABTS Assay

**Figure 3:**
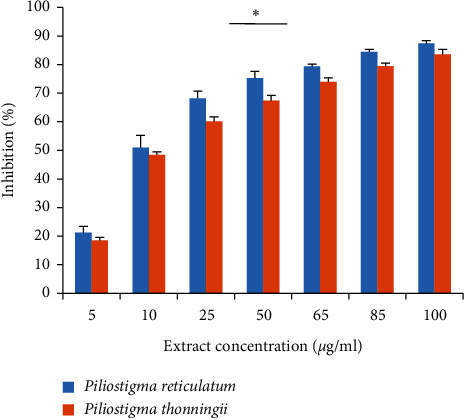
ABTS scavenging ability of *P. reticulatum* and *P. thonningii* methanolic leaves extracts. The kinetics of the two extracts is significantly different (*p* < 0.05).

The ABTS scavenging ability results of *P. reticulatum* and *P. thonningii* leaves are presented in Figure 3. For both plants, an antioxidant activity was noticed for all concentrations. At 25 *μ*g/mL, the methanol leaves' extracts of *P. reticulatum* and *P. thonningii* start showing a significant scavenging ability, which reached 68.21% and 60.13%, respectively. The inhibition kept rising and reached 87.42% for *P. reticulatum* and 83.63% for *P. thonningii* at the concentration of 100 *μ*g/mL. The IC_50_ values for *P. reticulatum* and *P. thonningii* methanol leaves extracts were 9.78 ± 1.83 *μ*g/mL and 13.47 ± 2.62 *μ*g/mL, respectively, and were statistically comparable and significantly higher than the IC_50_ of Trolox (30.76 ± 0.18 *μ*g/ml) (Table 3).

### 3.4. In Vitro Anti-Inflammatory Activity

**Figure 4:**
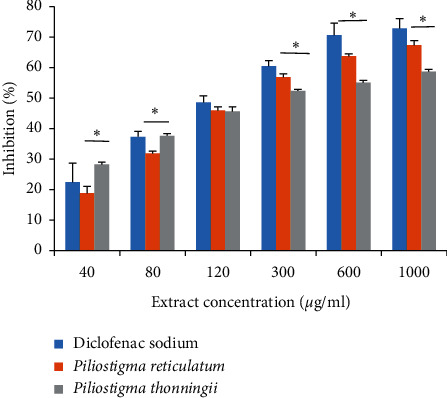
Effect of *P. reticulatum* and *P. thonningii* leaves extracts on heat-induced albumin denaturation in comparison with diclofenac sodium activity. *P. thonningii* inhibition ability is significantly low (*p* < 0.05).

The inhibition of thermally induced protein denaturation protocol was used to assess the anti-inflammatory effect of the extracts. For this, various concentrations (40 to 1000 *μ*g/mL) of the two plants' methanol leaves extracts were compared with the same concentrations of the reference drug, diclofenac sodium, as presented in Figure 4. At the lowest concentrations, *P. thonningii* had the highest inhibitory effect, which reached 37.68%. From 120 *μ*g/mL, the standard has shown a significant increase in its anti-inflammatory activity. This increase reached 72.82% at the maximal concentration, exceeding the two plants' activities (58.69% for *P. reticulatum* and 67.39% for *P. thonningii*). There is no statistically significant difference between the kinetics of *P. reticulatum* and diclofenac, whereas the activity of *P. thonningii* is relatively low. The standard has a high IC_50_ value (116.4 ± 0.73 *μ*g/mL) compared to *P. reticulatum* (121.43 ± 1.55 *μ*g/mL) and *P. thonningii* (170.15 ± 1.09 *μ*g/mL) (Table 4).

## 4. Discussion

Many plants produce a multitude of secondary metabolites to ensure their protection, communication, and adaptation to the environment. The diversity of these metabolites, endowed with structural variability, is responsible for many bioactive properties of these plants [22]. This study focuses on the medicinal value of two Sub-Saharan plants; *P. reticulatum* and *P. thonningii*, which lies particularly in their richness in secondary metabolites. In fact, all parts of both plants (roots, bark, pods, and leaves) seem to have therapeutic properties and therefore are used in traditional medicine. In the present study, *P. reticulatum* and *P. thonningii* were evaluated for their phenols and sugars content, antioxidant activity, and anti-inflammatory effect. The leaves of the two plants were dried and then extracted using solvents with increasing polarity (petroleum ether, dichloromethane, dichloromethane/methanol (80/20), and methanol); four extracts of different yields and colors were obtained. Phytochemical screening was performed to reveal the molecules' families contained in the leaves of both plants. The methanolic extracts were then selected for further testing. The determination of polyphenols and sugars concentration was first carried out and was followed by the evaluation of antioxidant and anti-inflammatory activities. Yields of leaves' extracts from both plants are statistically comparable for each plant. Methanolic extracts represent the highest yield for both plants. Indeed, the extraction power of the solvent has a significant influence on the yield [23]. This result can be explained by the fact that methanol is a polar solvent allowing interactions with ionic and polar solids, thus promoting their dissolution [24]. This can also be confirmed by the results of the phytochemical screening, which highlighted the richness of the leaves of both plants in different polar molecules mainly tannins, flavonoids, and alkaloids. Following these results, methanolic extracts were selected for the continuation of the study. The phytochemical assay demonstrated the richness of these two plants' leaves in polyphenols which explains their medicinal value. Indeed, flavonols and oxychromonol isolated from the leaves of *P. reticulatum* showed an interesting antimicrobial and antifungal activity [25]. Also, C-methylflavonols isolated from *P. thonningii* showed a strong anti-inflammatory and antibacterial effect [26]. The DPPH test and ABTS assay were used to evaluate the antioxidant activity of the leaf extracts of both plants. This study's results showed a significant divergence of the two plants' antiradical power, so that *P. reticulatum* has the highest activity. Also, the tannins antioxidant power was demonstrated, via the precipitation of the condensed tannins contained in the plant [27]. Nevertheless, the antioxidant activity of *P. thonningii* remains significant. In fact, the in vivo study of the antioxidant effect of *P. thonningii* leaves has shown that they can protect the liver from carbon tetrachloride caused oxidative damage via free radicals scavenging and lipid peroxidation actions [28]. The test of the inhibitory potential of our extracts on heat-induced denaturation of albumin was carried out to evaluate their anti-inflammatory effect. In fact, C-methylflavonols, isolated from the *P. thonningii* leaves, have already been shown to be more effective than aspirin against the in vitro production of prostaglandin [22], and the fact that they are also able to prevent protein denaturation confirms the similitude of their anti-inflammatory mode of action to nonsteroidal anti-inflammatory drugs (NSAIDs) [20]. Indeed, NSAIDs have been shown to inhibit, not only the synthesis of proinflammatory prostaglandins but also proteins denaturation [29], such as albumin denaturation, thus preventing the production of autoantigens. The anti-inflammatory effect of *P. reticulatum* bark against carrageenan-induced oedema of the rat hind leg has recently been evaluated, and the methanolic fraction had the same effect as salicylic acid [30], showing that the anti-inflammatory effect of this plant is not limited to its leaves.

## 5. Conclusion

Because of their richness in flavonoids and tannins, *P. thonningii* and *P. reticulatum* have shown a very interesting antioxidant and anti-inflammatory potential making these plants remarkable candidates for conventional medicine. Our study results are promising and encourage us to deepen our investigation by performing in vivo and toxicity tests for a better understanding of these plants extracts mode of action and the probable limits of their use.

## Figures and Tables

**Table 1:** Results of phytochemical screening of the composition of *Piliostigma reticulatum* and *Piliostigma thonningii* leaves.

	Petroleum ether		DCM		DCM/methanol		Methanol	
	*P. thonningii*	*P. reticulatum*	*P. thonningii*	*P. reticulatum*	*P. thonningii*	*P. reticulatum*	*P. thonningii*	*P. reticulatum*
Polyphenols	+	+	+	+	+++	++	+++	+++
Tannins	−	−	+	+	++	++	+++	++
Flavonoids	−	+	+	+	++	++	++	++
Alkaloids	−	−	−	+	+	++	++	++
Sterols	+	−	−	−	−	−	−	−
Terpenoids	−	−	−	+	−	+	−	+
Saponosides	−	−	+	−	+	−	+	−

−: not detected; +: rare; ++: abundant; +++: very abundant.

**Table 2:** The total phenolic-sugar contents in *P. reticulatum* and *P. thonningii* methanolic leaves extracts.

	*P. thonningii*	*P. reticulatum*
Total polyphenols (GAE *μ*g/mL)	56. 54 ± 1. 24	74.66 ± 1.76 ^*∗∗*^
Total tannins (CE *μ*g/mL)	33.87 ± 1.09	58. 28 ± 0.94 ^*∗∗∗*^
Total flavonoids (QE *μ*g/mL)	21. 59 ± 0.75	13. 57 ± 2.93 ^*∗∗*^
Total sugars (GE *μ*g/mL	13.34 ± 1.82	71.49 ± 0.57 ^*∗∗∗∗*^

Values are mean ± SD of triplicate readings (*n* = 3). The results are significantly different for *p* < 0.05. ^*∗∗*^0.001 < *p* < 0.01; ^*∗∗∗*^0.0001 < *p* < 0.001; ^*∗∗∗∗*^*p* < 0.0001.

**Table 3:** DPPH IC_50_ and ABTS IC_50_ values of the two plants extracts and their standards.

	*P. thonningii*	*P. reticulatum*	Ascorbic acid	Trolox
DPPH IC_50_ (*μ*g/mL)	17.64 ± 0.684 ^*∗∗∗∗*^^a^^*∗∗∗∗*^^b^	8. 88 ± 0.114 ^*∗∗∗∗*^^b^	1.745 ± 0. 28	-
ABTS IC_50_ (*μ*g/mL)	9.78 ± 1, 836^ns a^^*∗∗∗∗*^^b^	13.47 ± 2.623 ^*∗∗∗∗*^^b^	-	30.769 ± 0.180

Values are mean ± SD of triplicate readings (*n* = 3). The results are considered significantly different for *p* < 0.05. ^ns^*p* ≥ 0.05 (no significant difference) ^*∗∗∗∗*^*p* < 0.0001; ^a^statistically different as compared to the other plant's extract; ^b^statistically different as compared to the standard.

**Table 4:** Albumin denaturation inhibition IC50 values of the two plants' extracts and diclofenac sodium.

	*P. thonningii*	*P. reticulatum*	Diclofenac sodium
IC_50_ (*μ*g/mL)	170.15 ± 1.09 ^*∗∗∗∗*^^a^^*∗∗∗∗*^^b^	121.43 ± 1.55 ^*∗∗*^^b^	116.4 ± 0.73

Values are mean ± SD of triplicate readings (*n* = 3). The results are considered significantly different for *p* < 0.05. ^*∗∗*^0.001 < *p* < 0.01; ^*∗∗∗∗*^*p* < 0.0001; ^a^statistically different as compared to the other plant's extract; ^b^statistically different as compared to the standard.

## Data Availability

The data used to support the findings of this study are available upon request.
